# Exploring workplace violence on surgical wards in Sweden: a cross-sectional study

**DOI:** 10.1186/s12912-023-01275-z

**Published:** 2023-04-07

**Authors:** Jenny Jakobsson, Karin Örmon, Malin Axelsson, Hanne Berthelsen

**Affiliations:** 1 Department of Care Science, Faculty of Health and Society, Malmö University, Malmö, Sweden; 2 Centre for Work Life and Evaluation Studies (CTA), Malmö University, Malmö, Sweden; 3 The Västra Götaland Region Competence Center on Intimate Partner Violence, Gothenburg, Sweden; 4 Faculty of Odontology, Malmö University, Malmö, Sweden

**Keywords:** Assistant nurse, Hospital organization, Questionnaire, Registered nurse, Surgical ward, Workplace violence

## Abstract

**Background:**

Workplace violence is a global threat to healthcare professionals’ occupational health and safety and the situation has worsened during the COVID-19 pandemic. This study aimed to explore workplace violence directed against assistant and registered nurses working on surgical wards in Sweden.

**Methods:**

This cross-sectional study was conducted in April 2022. Using a convenience sampling procedure, 198 assistant and registered nurses responded to an online questionnaire developed for this specific study. The questionnaire comprised 52 items and included, among other items, subscales from validated and previously used instruments. Data analysis included descriptive statistics, the chi-square test, and independent-samples *t*-test.

**Results:**

The most frequently reported type of workplace violence was humiliation (28.8%), followed by physical violence (24.2%), threats (17.7%), and unwanted sexual attention (12.1%). Patients and patients’ visitors were reported as the main perpetrators of all kinds of exposure. Additionally, one third of the respondents had experienced humiliation from colleagues. Both threats and humiliation showed negative associations with work motivation and health (p < 0.05). Respondents classified as working in a high- or moderate-risk environment were more frequently exposed to threats (p = 0.025) and humiliation (p = 0.003). Meanwhile, half of the respondents were unaware of any action plans or training regarding workplace violence. However, of those who indicated that they had been exposed to workplace violence, the majority had received quite a lot or a lot of support, mainly from colleagues (range 70.8-80.8%).

**Conclusion:**

Despite a high prevalence of workplace violence, and especially of humiliating acts, there appeared to be low preparedness within the hospital organizations to prevent and/or handle such incidents. To improve these conditions, hospital organizations should place more emphasis on preventive measures as part of their systematic work environment management. To help inform such initiatives, it is suggested that future research should focus on the identification of suitable measures regarding different types of incidents, perpetrators, and settings.

## Background

According to the World Health Organization (WHO), healthcare professionals’ occupational health and safety are fundamental prerequisites for a sustainable work life, and a well-functioning healthcare system [1]. Still, it has been reported that as many as 62% of healthcare workers have experienced workplace violence (WPV) [2]. Moreover, the COVID-19 pandemic has worsened the situation because it has added further pressure and risk to an already strained workforce [3]. The vast majority of previous research has focused on the prevalence of WPV. However, to be able to create appropriate safety measures to prevent and/or handle incidents, there is a need to identify situations that could trigger WPV, who is at risk, who the perpetrators are, and to investigate organizational preparedness to prevent and/or handle such events. This study attempted to investigate these questions.

Workplace violence has been referred to as: “Incidents where staff are abused, threatened or assaulted in circumstances related to their work, including commuting to and from work, involving an explicit or implicit challenge to their safety, well-being or health” [4; p.3]. During the last few decades, numerous studies have investigated WPV directed against healthcare professionals. The wealth of studies became apparent in a systematic review and meta-analysis conducted by Liu et al. [2], which included 253 studies published during the last 30 years reporting on the prevalence of WPV. This number of studies implies that WPV arouses great interest, but may also indicate a need to look beyond prevalence rates and delve more deeply into the problem. This was done, for example, by Perkins et al. [5] and Babiarczyk et al. [6]. In their studies, patients were mentioned as being behind the majority of both physical assaults and verbal abuse, although patients’ relatives were also reported as responsible for almost half of the verbal abuse. Bigham et al. [7] further elaborated on the type of incidents and found that physical assaults, mostly undertaken by patients, included for example kicking, hitting, biting, or slapping. Verbal abuse was attributed to both patients and their relatives and included the use of offensive language, criticism, or threats of violence. Sexual harassment also occurred, in the form of joking, making obscene gestures, or using derogatory epithets. However, there have been perpetrators other than patients or relatives reported in previous research; for example, other staff members, or managers/supervisors [6]. In a multicenter study by La Torre et al. [8], as many as 31.5% of participants reported attacks from another healthcare worker. This lateral violence has been defined as “repeated, offensive, abusive, intimidating, or insulting behavior, abuse of power, or unfair sanctions that makes recipients upset and feel humiliated, vulnerable, or threatened, creating stress and undermining their self-confidence” [9; p.136]. Considering the wealth of studies investigating WPV against healthcare professionals, it can be argued that lateral violence has received less attention than patient- or relative-induced violence. Nonetheless, it needs to be equally acknowledged since it has been shown to be correlated with burnout, lower job satisfaction, and reduced general health among exposed individuals [10, 11].

The risk of being exposed to WPV has been shown to be related to the healthcare context and its organizational features. Both physical and psychological violence have been most frequently reported within prehospital care, in emergency departments, and in psychiatric and geriatric care [5, 6]. However, this does not mean that there is an absence of WPV on general hospital wards. On the contrary, a high prevalence has been found within different hospital settings, and especially in surgical, medical, and intensive care units [12, 13]. But, in contrast to prehospital care and emergency departments, it is common for patients on general hospital wards to be hospitalized for longer periods. Thus, the healthcare professionals caring for them will be at prolonged risk of exposure to WPV when interacting with a potentially abusive patient or relative. On surgical wards, staff are caring for patients who may be cognitively affected due to an underlying disease, for example dementia, a gastrointestinal infection, or related to trauma, surgery, or medication with opioids. Any of these can result in physically and/or verbally aggressive behavior [14]. Hence, healthcare professionals who work on surgical wards face a considerable risk of being exposed, but few studies have addressed WPV in this specific context [15, 16].

Other studies have proposed additional factors increasing the risk of exposure among healthcare workers in general. For example, working shifts—and especially night shifts—working more than 40 h per week, or being of younger age [5, 8, 17]. It could be argued that such factors are inherent features of healthcare work and, therefore, the associated risks should be of high priority within these organizations. Furthermore, it has been shown that in hospital organizations with a poor psychosocial safety climate (PSC), i.e. where the prevention of risks within the work environment is of low priority, there is a higher prevalence of WPV [18]. Healthcare professionals working on surgical wards have described how physical violence and verbal abuse made them feel scared and unprotected, but that inadequate preventive strategies induced a perception that WPV should be tolerated as part of the job [15]. This finding further suggests that there might be aspects within the healthcare organizations’ PSC that are related to an increased risk of WPV, but this has not been sufficiently addressed in a surgical context before. Moreover, it has been recommended that, in future research, the PSC should be included as a determinant of psychosocial work hazards [18].

In hospitals, there is a high prevalence of WPV. This can lead to a deteriorating work environment that threatens professionals’ safety, health, and work motivation. In addition, earlier studies have indicated that hospital organizations might make insufficient efforts to prevent risks within the work environment. Since WPV includes different types of events and perpetrators, organizational safety measures need to be adapted to adequately address the incidents that occur. To gain comprehensive knowledge about WPV from a surgical in-hospital context, the overall purpose of this study was to explore WPV directed against registered nurses and assistant nurses working on surgical wards in Sweden. More specifically, answers to the following research questions were sought:

i. What types of workplace violence occur on surgical wards and who are the typical perpetrators?
ii. How is exposure to the various types of WPV related to personal background characteristics (i.e. gender, and age), work characteristics (i.e. occupation, type of employment, and work experience), and organizational factors (i.e. PSC risk level)?
iii. To what extent is there an organizational preparedness for preventing and/or handling workplace violence?
iv. How does exposure to workplace violence relate to work motivation and health outcomes?

## Methods

### Design

This study had an explorative, cross-sectional design based on questionnaire data, and adhered to the guidelines: Strengthening the Reporting of Observational Studies in Epidemiology, STROBE [19].

### Setting and participants

It has been suggested that persons who work in close physical proximity to patients are at high risk of being exposed to different kinds of workplace violence [16]. Therefore, inclusion criteria were being an assistant nurse or registered nurse working on a surgical ward. Recruitment was conducted nationally using a convenience sampling procedure with the aim of including the widest possible number of respondents representing both larger and smaller cities.

### Data collection

To be able to access respondents for recruitment, permission to contact ward personnel was sought from the operations manager of all surgical departments in Sweden. Permission was granted by 30 hospitals located across 17 out of 21 regions. Due to travel restrictions during the COVID-19 pandemic, the researchers were unable to distribute the questionnaires in person as initially planned. Therefore, ward managers were asked to act as gatekeepers. Hence, an invitation to fill out the online questionnaire was composed by the research group and sent by e-mail to the ward managers, who then forwarded this invitation e-mail to assistant nurses and registered nurses on their wards. In this way, it was possible to invite assistant nurses and registered nurses working on a total of 53 surgical wards to answer the questionnaire. The e-mail contained written information about the study and a link and a QR-code to access the online questionnaire. Two reminders were sent out via the gatekeepers and the questionnaire was open for responses during April 2022.

### Instrument

An online questionnaire developed by the researchers for this specific study was used to collect data. The questionnaire comprised 41 questions with predefined response alternatives and 11 open questions. To address the first and second research questions, exposure to different kinds of WPV—i.e. threats of violence, physical violence, humiliation (in the questionnaire exemplified as for example being mocked, belittled, or humiliated), and unwanted sexual attention—were explored using selected items from the Swedish standard version of the Copenhagen Psychosocial Questionnaire (COPSOQ III) [20, 21]. Hence, respondents were asked to indicate if, and if so how often, they had been exposed to the specific issue during the previous twelve months. Response alternatives were *yes—every day, yes—every week, yes—every month, yes—occasionally*, and *no*. For analysis, the responses were dichotomized into *yes* and *no*. Following these questions, respondents could indicate who the perpetrator was with context-specific response options developed for the current study (these differed from the COPSOQ III response options): *patients*, *patients’ visitors*, *superiors*, *colleagues*, and *other staff members*. To further develop these answers, open questions then allowed respondents to write in free format about their experience and if they had told anyone about it [22].

To explore the exposure to different kinds of WPV in relation to personal background characteristics and work characteristics, questions about details such as gender, age, occupation, and type of employment were asked. Regarding organizational factors, the Psychosocial Safety Climate (PSC) short version was used (4 items) as an indicator of employees’ perception of the extent to which their psychological health and safety are given priority within the hospital organization [23, 24]. Items from the PSC could be answered on a 5-point Likert scale and were scored from 1 (strongly disagree) to 5 (strongly agree), thus allowing a total score in the range from 4 to 20. Scale scores were calculated as the mean item score and were set as “missing” if respondents had answered fewer than two items. Based on these scale scores, respondents were classified into three PSC risk groups: high, moderate, and low [23].

To answer the third research question, participants’ awareness of preventive measures and action plans were explored using questions that were developed based on results from previous studies describing experiences of WPV [15, 16]. For example: *“Is there any training at your workplace about threats and violence?”* Response alternatives were *yes*, *no*, and *unsure*. For this specific question, respondents could specify the type of training by describing it in free writing. Respondents who had experienced any kind of WPV were also asked to answer questions about perceived support and how they had been affected by the incident. Response options ranged from *not at all* to *very much*.

The last research question was explored by using four scales from the COPSOQ III instrument [20, 21]. These four scales were selected for the operationalization of *Commitment to the workplace* (3 items), *Quality of work* (2 items), *Work engagement* (3 items), and *Stress* (3 items). Proprietary items were added measuring *Symptoms of distress* (3 items) and *Sleeping problems* (2 items). All these items could be answered on a 5-point Likert scale ranging from *a very small extent* to *a very large extent*, or from *never* to *always* depending on how the items were formulated. Items were scored as 0–25 – 50–75 – 100 following the direction of the response alternatives. Scale scores were calculated as the mean item score and were set as “missing” if respondents had answered fewer than half of the questions in the scale [21].

Internal consistency regarding scales adopted from the COPSOQ III and PSC was tested during analysis using Cronbach’s alpha and showed to be satisfactory (*Commitment to workplace* α = 0.80, *Quality of work* α = 0.71, *Work engagement* α = 0.75, *Stress* α = 0.84, *PSC scale* α = 0.91). The same was shown for the proprietary items (*Sleeping problems* α = 0.83 and *Symptoms of distress* α = 0.79).

### Data analysis

The data was analyzed using IBM SPSS Statistics version 28. Descriptive statistics were used to present the study sample. Frequencies of exposure to WPV during the previous 12 months were calculated according to personal background (i.e. gender, age), work characteristics (i.e. occupation, type of employment, work experience, work schedule) and organizational factors (i.e. PSC risk level). The distribution within each group was tested using the Chi-square test. Next, for those respondents who reported having been exposed, the frequencies of different groups of perpetrators, support received, and perceived impact were calculated in relation to each type of WPV. Finally, mean scores and standard deviations (SD) for *Commitment to the workplace*, Q*uality of work*, *Work engagement*, *Stress*, *Symptoms of distress* and *Sleeping problems* were calculated for each type of WPV, and then compared between exposed and non-exposed respondents using independent samples *t*-tests.

The number and proportion of respondents providing free-writing responses about their experiences in the open questions were calculated by manually counting the responses. Because respondents only wrote short phrases containing single words, an in-depth analysis was not relevant. Hence, a brief content analysis was conducted to compile the information provided in the responses [22].

## Results

Of the 198 respondents, the majority were registered nurses (58.1%), women (89.9%), and working full time (76.3%). Mean age was 41 years (SD 12 years, range 20–68 years). Respondents reported that they had worked within the healthcare sector for an average of 14 years (SD 11 years, range 0–50 years). They had been working on their current ward for a mean of 7 years (SD 7 years, range 0–32 years). Further details are presented in Table 1.

**Table 1:** Background characteristics of respondents (n = 198)

Characteristics	n (%)
Gender	
Woman	178 (89.9)
Man	17 (8.6)
Missing	3 (1.5)
**Age group**	
20–29 years	34 (17.2)
30–39 years	69 (34.8)
40–49 years	42 (21.2)
50–59 years	39 (19.7)
60–69 years	13 (6.6)
Missing	1 (0.01)
**Occupation**	
Assistant nurse	81 (40.9)
Registered nurse	115 (58.1)
Missing	2 (1.0)
**Employment**	
Full time	151 (76.3)
Part time	46 (23.2)
Missing	1 (0.01)
**Experience in healthcare**	
<7 years	63 (31.8)
7–15 years	65 (32.8)
>15 years	70 (35.4)
Missing	0 (0.00)
**Work schedule, shift work** *(Multiple responses possible)*	
Day	168 (84.8)
Evening	136 (68.7)
Night	68 (34.3)
Missing	1 (0.01)
**Work schedule weekdays**	
Mainly weekdays	29 (14.6)
Mixed weekdays and weekends	166 (83.8)
Missing	3 (1.5)
**Psychosocial Safety Climate**	
High PSC-risk (PSC < 8)	63 (31.8)
Moderate PSC-risk (PSC > 8–12)	79 (39.9)
Low PSC-risk (PSC > 12)	47 (23.7)
Missing	9 (4.5)

n = number

### Various types of WPV and typical perpetrators

The most frequently reported exposure to WPV during the previous 12 months was humiliation, which was reported by 28.8% of all respondents, followed by physical violence (24.2%), threats (17.7%), and unwanted sexual attention (12.1%) (Table 2). Patients were the most frequently reported perpetrators of all four types of WPV included in the questionnaire. Patients’ visitors and professional colleagues were also described as responsible for threats, humiliation, and unwanted sexual attention, as further detailed in Table 3.

**Table 2:** Number and proportion of respondents exposed to WPV and differences with regard to subgroups

Subgroup	Threats (n = 198) n (%)	Physical violence (n = 197) n (%)	Humiliation (n = 198) n (%)	Unwanted sexual attention (n = 197) n (%)
**All**	35 (17.7)	48 (24.2)	57 (28.8)	24 (12.1)
**Gender**				
Woman	30 (16.9)	42 (23.7)	52 (29.2)	22 (12.4)
Man	5 (29.4)	6 (35.3)	4 (23.5)	1 (5.9)
*p*-value	0.389	0.485	0.723	0.679
**Age group**				
20–29 years	6 (17.6)	6 (17.6)	11 (32.4)	5 (14.7)
30–39 years	12 (17.4)	18 (26.1)	21 (30.4)	13 (18.8)
40–49 years	9 (21.4)	10 (23.8)	13 (31.0)	3 (7.1)
50–59 years	6 (15.4)	12 (30.8)	10 (25.6)	3 (7.7)
60–69 years	2 (15.4)	2 (15.4)	2 (15.4)	0 (0.0)
*p*-value	0.964	0.630	0.787	0.166
**Occupation**				
Assistant nurses	12 (14.8)	22 (27.2)	18 (22.2)	10 (12.3)
Registered nurses	22 (19.1)	26 (22.6)	39 (33.9)	13 (11.3)
*p*-value	0.432	0.435	0.076	0.799
**Employment**				
Full time	27 (17.9)	33 (22)	43 (28.5)	18 (12.0)
Part time	8 (17.4)	14 (30.4)	14 (30.4)	6 (13.0)
*p*-value	0.939	0.241	0.798	0.850
**Experience in healthcare**				
<7 years	9 (14.3)	12 (19.0)	23 (36.5)	9 (14.3)
7–15 years	11 (16.9)	15 (23.1)	16 (24.6)	9 (13.8)
>15 years	15 (21.4)	21 (30.4)	18 (25.7)	6 (8.7)
*p*-value	0.549	0.301	0.258	0.545
**Work schedule weekdays**				
Mainly weekdays	4 (13.8)	2 (6.9)	7 (24.1)	3 (10.3)
Mixed weekdays and weekends	31 (18.7)	44 (26.7)	50 (30.1)	21 (12.7)
*p*-value	0.527	**0.021**	0.513	0.719
**Psychosocial Safety Climate**				
High PSC risk	18 (28.6)	19 (30.2)	25 (39.7)	10 (16.1)
Moderate PSC risk	11 (13.9)	16 (20.3)	25 (31.6)	8 (10.1)
Low PSC risk	5 (10.6)	10 (21.3)	5 (10.6)	6 (12.8)
*p*-value	**0.025**	0.347	**0.003**	0.570

n = number. *p*-values < 0.05 was considered significant and is market using bold text. Differences between subgroups were calculated using Chi-square test

**Table 3:** Proportions (%) of reported perpetrators among respondents exposed to a specific WPV.

	Patient	Visitor	Superior	Colleague	Other staff member
Threats (n = 35)	85.7	22.9	5.7	5.7	2.9
Physical violence (n = 48)	97.9				
Humiliation (n = 57)	64.9	29.8	3.5	31.6	8.8
Unwanted sexual attention (n = 24)	79.2	0.0	0.0	25.0	4.2

Note: More than one kind of perpetrator could be reported (except in relation to physical violence where a technical error blocked for this)

n = Total number of respondents reporting the specific exposure

### Responses to open questions describing type of WPV and perpetrator

In the open questions, several respondents used the opportunity to further elaborate upon their specific experiences. Threats were further described by 27 respondents (77% of those who had experienced threats). Their experiences mostly consisted of verbal threats made by both patients and their visitors. Examples included threats of violence, the use of swear words, or yelling. There were also experiences of patients or visitors acting in a threatening manner.

Physical violence was further described by 44 respondents (92% of those who had been exposed to physical violence). The most common perpetrator was described as a patient with dementia. Incidents included punches, pinches, scratches, kicks, attempts to kick, being held tightly in the arms, or when patients threw things after the healthcare professionals.

Humiliation was enacted by a variety of individuals, according to the 42 respondents who further elaborated upon this type of WPV (74%). Patients or relatives who were upset or dissatisfied were mentioned as perpetrators, but also colleagues, doctors, or superiors. Abusive and derogatory comments were the most commonly experienced form of humiliation. For example, patients or relatives might question respondents’ competence or suitability to work within healthcare. Some had experienced verbal violations based on their ethnicity, physical appearance, or youth. Humiliation from colleagues, doctors, or superiors mostly involved speaking badly about someone, or making unkind and disparaging comments.

Unwanted sexual attention was further explained by 23 respondents (96%) as stemming mostly, but not exclusively, from patients. These incidents comprised sexually suggestive comments or sexual touches. One respondent had experienced sexually suggestive comments from a colleague in a more senior position.

For all kinds of exposure, the majority of respondents stated that they had told someone (ranging from 66.7% for unwanted sexual attention to 91.7% for physical violence). Almost all respondents who had been exposed to any kind of incident specified that they had told a colleague. Regarding threats and physical violence, about half of the respondents specified that they had told their ward manager or another superior. For humiliation and unwanted sexual attention, this proportion was lower, with the results showing that only one third had reported it.

### Exposure to WPV in relation to personal background characteristics, work characteristics, and organizational factors

Differences in exposure broken down by subgroups of respondents are presented in Table 2. Physical violence was significantly more frequently reported by respondents working a mixture of weekdays and weekends compared to those who worked mainly on weekdays. Threats and humiliation were reported less frequently by respondents classified as working in a low-PSC-risk environment than by those working in environments with higher risk.

### Organizational preparedness for preventing and/or handling WPV

**Fig. 1:**
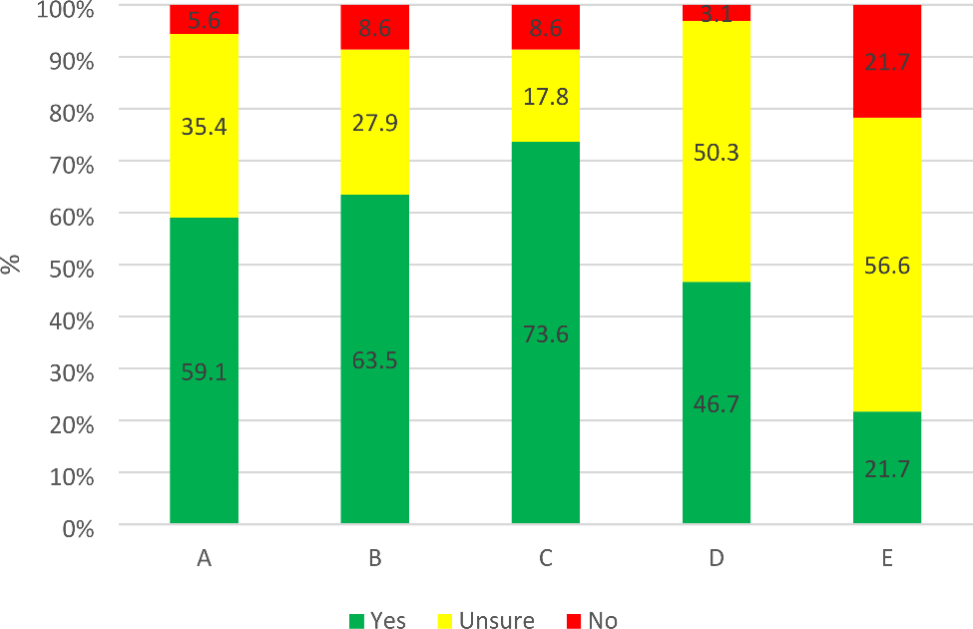
Distribution of respondents’ replies concerning organizational preparedness for preventing and handling WPV. Specific questions: A: If you are exposed to threats or violence from a patient or visitor, do you know how to handle the situation in practice? (n = 198) B: If you are exposed to threats or violence from a patient or visitor, do you feel that you could handle the situation emotionally? (n = 197) C: Do you know who or where to turn if you are exposed to threats or violence in your workplace? (n = 197) D: Do you have action plans for threats and violence in your workplace? (n = 195) E: Is there any training about threats and violence in your workplace? (n = 198)

Although most respondents reported being confident about how to handle WPV, around a third felt unsure if they could handle it (Fig. 1). Moreover, more than half of the respondents did not know if there were any action plans or training in the workplace. Among those who stated that they had any form of training, most reported it to be a web-based education.

Among respondents who had experienced one or more incident during the previous year, the majority had received *quite a lot* or *a lot* of support. Colleagues were the most frequent source of support for all kinds of incidents (Table 4). Furthermore, 14% reported not having received any support at all from their ward manager (result not shown in the table). In general, fewer than 10% of those who had been exposed felt that it had influenced them *quite a lot* or *a lot* in their professional role or as a private person.

**Table 4:** Proportions (%) of respondents exposed to WPV during the previous 12 months (n = 101) and indicating quite a lot/a lot to questions about received support and impact on different aspects of life

	Proportions (%) indicating quite a lot/a lot among respondents exposed to:			
*Question: When you have been subjected to threats or violence, to what extent do you feel that you have…*	Threats	Physical violence	Humiliation	Unwanted sexual attention
- received support from your immediate superior? (n = 92)	54.3	66.6	59.1	47.8
- received support from colleagues? (n = 94)	71.4	80.8	74.2	70.8
- been affected in relation to your professional role? (n = 99)	8.6	4.2	7.3	8.3
- been affected in relation to you as a private person? (n = 98)	5.8	7.8	5.4	4.2
-been affected in relation to your health? (n = 99)	14.3	8.3	14.5	12.5

n = number of respondents exposed to WPV

### Exposure to WPV and its relation to work motivation and health

Respondents who had experienced humiliation during the previous 12 months reported significantly lower levels of *commitment to their workplace*, *quality of the work* performed at their workplace, and *work engagement*, as well as higher levels of *stress, symptoms of distress*, and *sleeping problems* compared to unexposed respondents. A corresponding tendency was also seen in relation to the other kinds of exposure, although in most cases these did not attain statistical significance. For further details, see Table 5.

**Table 5:** Factors related to work motivation and health in relation to exposure to different kinds of WPV.

	Threats			Physical violence			Humiliation			Unwanted sexual attention		
	No (n = 163)	Yes (n = 35)		No (n = 149)	Yes (n = 48)		No (n = 141)	Yes (n = 57)		No (n = 173)	Yes (n = 24)	
	Mean (SD)	Mean (SD)	*p*	Mean (SD)	Mean (SD)	*p*	Mean (SD)	Mean (SD)	*p*	Mean (SD)	Mean (SD)	*p*
Commitment to workplace*	69.4(19.0)	55.0(23.2)	**< 0.001**	68.7(20.7)	60.8(18.8)	**0.019**	70.3(18.7)	58.2(22.2)	**< 0.001**	67.6(20.6)	61.8(19.3)	0.193
Quality of work*	70.8(15.4)	61.8(17.1)	**0.002**	70.3(15.4)	65.6(17.9)	0.079	71.8(15.3)	62.7(16.1)	**< 0.001**	69.9(16.1)	65.1(14.7)	0.172
Work engagement*	75.9(14.5)	70.6(14.8)	0.052	76.1(14.6)	71.8(14.5)	0.081	77.0(14.2)	70.2(14.6)	**0.003**	75.0(14.5)	76.4(14.4)	0.659
Stress*	44.9(22.6)	52.7(23.4)	0.070	44.6(23.3)	50.5(20.5)	0.121	41.7(21.9)	57.5(21.4)	**< 0.001**	45.6(22.5)	51.1(25.4)	0.278
Symptoms of distress*	31.4(21.1)	45.3(22.8)	**< 0.001**	33.3(21.9)	35.6(22.6)	0.520	29.5(20.4)	44.4(22.4)	**< 0.001**	33.1(21.2)	39.1(23.9)	0.220
Sleeping problems*	41.3(24.4)	48.2(26,3)	0.145	40.9(25.2)	48.1(23.0)	0.080	39.1(24.3)	50.9(24.3)	**0.002**	42.1(24.3)	45.7(29.6)	0.516

Mean scale scores and standard deviations (SD) are visualized for scales measuring relation to work motivation and aspects of health

with regard to non-exposure (No) or exposure (Yes) to different kinds of WPV.

*p* < 0.05 was considered as a significant difference. Significant values are indicated using bold text

*Range 0-100

## Discussion

This study essentially showed that assistant nurses and registered nurses on surgical wards in Sweden experience very high exposure to WPV, with potentially negative consequences for their work motivation and health. The study also identified humiliation as the most common form of incident, with not only patients and relatives, but also colleagues, being frequently involved as perpetrators. The majority of respondents were classified as working in a moderate- or high-risk environment, which was also related to exposure to threats and humiliation. In the following, these major findings will be discussed.

When exploring various types of WPV, the results show that humiliation was the most common form of incident, reported by almost one third of respondents. Such humiliation was related to reduced work motivation, stress, symptoms of distress, and sleeping problems. The humiliation was inflicted mostly by patients, but at times also by visitors and colleagues. Respondents explained that patients and visitors may make abusive and derogatory comments or verbal violations based on personal attributes. Similar results have been reported in previous studies. Respondents in a study by Rosenthal et al. [25] indicated that 10% of all verbal harassment perpetrated by patients or their relatives was based on race, gender, or sexual identity and, according to the respondents in Jakobsson et al. [15], gender discrimination led to feelings of disrespect and degradation. Furthermore, in the current study humiliation from colleagues was described as including speaking badly about each other or making unkind or disparaging comments. Other studies have designated this problem as lateral violence, but have reported a much higher prevalence. For example, in a study involving nurses in a public hospital in Spain, more than half of the respondents had experienced lateral violence from co-workers. This was enacted by spreading false rumors or ignoring the respondents [10]. Another study, from the United States, confirmed a high frequency of lateral violence, with a prevalence of 40.1%. In that study, negative associations with resilience, physical health, and mental health were also reported [26]. Even though lateral violence from colleagues was less frequent in the current study than in other studies, it is a form of WPV that has negative consequences for exposed persons. Thus, it cannot be ignored as a work environment problem. It can even be suggested that lateral violence might put more strain on the individuals exposed than does verbal abuse from patients or patients’ visitors. This is because patients are likely to be discharged once they have recovered, while colleagues will continue to be present on an everyday basis. Therefore, there should be a zero-tolerance policy within hospital organizations regarding lateral violence. However, it has been observed that healthcare professionals who are subjected to WPV in general do not always report it to their immediate superior [16]. This leads to difficulties in addressing the problem, regardless of the type of event involved. Similar results have been reported in another study, which also explained that healthcare professionals refrained from reporting incidents because they did not believe that it would lead to any changes [27]. The results of the current study could not confirm these previous findings because the vast majority of exposed respondents stated that they had told someone about it. Although the majority of respondents stated that they had told colleagues and received most support from them, they had also frequently told their ward manager or another superior and received almost the same degree of support from them as from colleagues.

After humiliation in frequency, exposure to physical violence was reported by a fourth of the respondents in the current study, followed by threats and unwanted sexual attention. This is a considerably higher prevalence than that reported by the Swedish working population in general [20]. In comparison, the most prevalent exposure in the Swedish workforce is to threats of violence (10.5%), followed by sexual harassment (6.0%), and lastly physical violence (5.3%) [20]. Thus, our findings indicate a noteworthy higher exposure to WPV among this sample of surgical nurses than among employees in general in Sweden. On the other hand, the exposure to physical violence and sexual harassment was at the same level as that reported by healthcare professionals internationally [3], although threats were less common in the current study. As stated earlier, the COVID-19 pandemic has worsened the situation for several reasons [3] and a recent systematic review and meta-analysis estimated that the prevalence of WPV during the COVID-19 pandemic was 47% [28]. Non-physical violence was the most commonly reported form of incident. Although, this higher frequency of non-physical violence compared to other forms of incidents has been reported both within the Swedish workforce and in studies published before the pandemic. For instance, in a study determining the prevalence of WPV against healthcare workers in an emergency setting, the prevalence of non-physical violence amounted to 73.1% [29].

There could be several explanations for the higher prevalence of WPV found in healthcare settings. Patients who receive care in hospitals have a variety of diseases or injuries that can influence their cognition. Moreover, patients’ relatives and visitors are likely to care about and protect their loved ones, and incidents may be an expression of frustration or worry. As an example, it has been reported that one major cause of violent acts was delays in receiving care [30]. Hence, it is already known and not a surprise that WPV occurs more frequently within healthcare than in other professions. But this also indicates that hospital organizations need to prevent predictable events and put in place sufficient measures to deal with events that will—inevitably—arise.

Considering the assumption that WPV is to be somewhat expected on a surgical ward, together with the fact that not *all* respondents in the current study stated that they had told their ward manager or received support from them, this might suggest that there could be organizational shortcomings in the prevention of work hazards. An additional indication of this could be the circumstance that more than half of the respondents were unaware of any education or routines related to WPV. On the PSC scale, respondents’ perception that their health and safety were of priority to the hospital organization [23] was measured. Hence, higher PSC risk indicates a preventive failure, but may also identify a potential area for improvement. The PSC risk was classified as high or moderate by the majority of respondents (31.8% and 39.9% respectively), while 23.7% worked in a low-risk environment. In comparison with population benchmarks, the PSC risk was greater in the current sample than for the general Swedish employee [23]. This result might suggest that the prevention of WPV is a deprioritized topic for some Swedish hospital organizations and, therefore, efforts need to be made to improve this condition. Aspects of the PSC within organizations have been highlighted in other studies before this. For example, despite an organizational zero-tolerance policy, participants in an interview study by Pich et al. [31] perceived that their employers’ strategies focused on reactions to violent behaviors instead of preventing them. This has also been described by participants in other studies [15, 32], in which participants experienced the lack of preventive strategies as signaling that they should tolerate WPV. In the current study, there was a higher prevalence of WPV compared to that found in general in the Swedish working population, together with considerably higher PSC risks [20, 23]. In the worst case, this could be interpreted as meaning that the hospital organizations actually do perceive WPV as an inherent part of the job. Otherwise, it could be argued that they would have taken noticeable actions to prevent incidents, which should lead to respondents indicating a lower PSC risk. On the other hand, as shown in this and previous research [16], superiors might be unaware of incidents because they are not reported to them. Knowing about the problem is a prerequisite for being able to prevent and/or handle incidents. This underlines the importance of systematic work environment management, including the creation of adequate reporting routines. It could also be argued that it is important to have a working climate in which professionals feel comfortable talking about the different incidents that can arise, and work together with each other and the hospital organization to promote preventive strategies as far as possible. This is especially important in order to minimize the risk of poor mental health and sickness absence, which are strongly associated with WPV [33].

### Limitations

Despite two reminders, only 198 assistant and registered nurses responded to the questionnaire, which could be considered a low number since the invitation was sent to 53 surgical wards, with two reminders. According to Nayak and Narayan [34], a poor response rate is a common issue when using online surveys although the response rate for this study is unknown since we do not know the total number of assistant and registered nurses working at the wards. Further, the sampling strategy did not allow for any non-response analysis. Thus, we cannot know the extent to which our findings can be generalized to a broader population and in the interpretation of the results, the risk of type I and type II errors should be considered. Nevertheless, the results, together with similarities with earlier research, indicate that despite a presumably low participation, the study had enough power to identify a number of interesting differences as well as cross-sectional relations.

## Conclusions

From this study, it can be concluded that, despite a very high prevalence of WPV, there appears to be a low preparedness for preventing and/or handling such incidents. This result is relevant to hospital organizations internationally because it highlights the importance of emphasizing preventive measures adapted to all the different types of workplace violence. Also, based on the current results, it is vital to emphasize that the reporting of incidents is a prerequisite for systematic work environment management. To facilitate this, adequate reporting routines need to be created so that all incidents can be acknowledged by the healthcare organization. Since there is already a large body of evidence demonstrating the prevalence of WPV, with all its related consequences, we suggest that future studies should focus on how different incidents could be prevented, as well as the impact of preventive measures on WPV prevalence and work-related health.

## Data Availability

The datasets used and analyzed during the current study are available from the corresponding author upon reasonable request.

## List of Abbreviations

COPSOQ: Copenhagen Psychosocial Questionnaire
PSC: Psychosocial Safety Climate
SD: Standard Deviation
STROBE: Strengthening the Reporting of Observational Studies in Epidemiology
WHO: World Health Organization
WPV: Workplace Violence

## References

[1] World Health Organization. : Protecting health and safety of health workers. https://www.who.int/activities/protecting-health-and-safety-of-health-workers. Accessed 13 Sep 2022.

[2] Liu J, Gan Y, Jiang H, Li L, Dwyer R, Lu K (2019). Prevalence of workplace violence against healthcare workers: a systematic review and meta-analysis. Occup Environ Med.

[3] ICN ICRC, IHF WMA. Violence against health care: Current practices to prevent, reduce or mitigate violence against health care. 2022. https://www.icn.ch/system/files/2022-07/Violence%20against%20healthcare%20survey%20report.pdf. Accessed 8 Sep 2022.

[4] World Health Organization: Framework Guidelines for Addressing Workplace Violence in the Health Sector, ILO/ICN/WHO/PSI Joint Programme on Workplace Violence in the Health Sector. (2002). https://www.who.int/publications/i/item/9221134466. Accessed 10 Nov 2022.

[5] Perkins M, Wood L, Soler T, Walker K, Morata L, Novotny A (2020). Inpatient Nurses’ perception of Workplace Violence based on Specialty. J Nurs Adm.

[6] Babiarczyk B, Turbiarz A, Tomagova M, Zelenikova R, Onler E, Cantus DS. Violence against nurses working in the health sector in five European countries-pilot study. Int J Nurs Pract. 2019;25(4). 10.1111/ijn.12744 31172630

[7] Bigham BL, Jensen JL, Tavares W, Drennan IR, Saleem H, Dainty KN (2014). Paramedic self-reported exposure to violence in the Emergency Medical Services (EMS) Workplace: a mixed-methods cross-sectional survey. Prehosp Emerg Care.

[8] La Torre G, Firenze A, Di Gioia LP, Perri G, Soncin M, Cremonesi D (2022). Workplace violence among healthcare workers, a multicenter study in Italy. Public Health.

[9] Vessey JA, Demarco R, DiFazio R (2010). Bullying, harassment, and horizontal violence in the nursing workforce: the state of the science. Annu Rev Nurs Res.

[10] Vidal-Alves MJ, Pina D, Puente-López E, Luna-Maldonado A, Luna Ruiz-Cabello A, Magalhães T et al. Tough love lessons: Lateral violence among hospital nurses. Int J Environ Res Public Health. 2021;18(17). 10.3390/ijerph18179183 PMC8431196 34501771

[11] Vessey JA, Williams L (2021). Addressing bullying and lateral violence in the workplace: a quality improvement initiative. J Nurs Care Qual.

[12] Hahn S, Zeller A, Needham I, Kok G, Dassen T, Halfens RJG (2008). Patient and visitor violence in general hospitals: a systematic review of the literature. Aggress Violent Behav.

[13] Spector PE, Zhou ZE, Xin Xuan C (2014). Nurse exposure to physical and nonphysical violence, bullying, and sexual harassment: a quantitative review. Int J Nurs Stud.

[14] Ferri P, Silvestri M, Artoni C, Di Lorenzo R (2016). Workplace violence in different settings and among various health professionals in an italian general hospital: a cross-sectional study. Psychol Res Behav Manag.

[15] Jakobsson J, Axelsson M, Örmon K. The Face of Workplace Violence: Experiences of Healthcare Professionals in Surgical Hospital Wards. Nurs Res Pract. 2020;e-collection 2020. 10.1155/2020/1854387 PMC7275198 32550024

[16] Jakobsson J, Örmon K, Berthelsen H, Axelsson M (2022). Workplace violence from the perspective of hospital ward managers in Sweden: a qualitative study. J Nurs Manag.

[17] D’Ettorre G, Pellicani V, Vullo A (2019). Workplace violence against healthcare workers in emergency departments. A case-control study. Acta Biomed.

[18] Pien L-C, Cheng Y, Cheng W-J (2019). Psychosocial safety climate, workplace violence and self-rated health: a multi-level study among hospital nurses. J Nurs Manag.

[19] von Elm E, Altman DG, Egger M, Pocock SJ, Gøtzsche PC, Vandenbroucke JP (2014). The strengthening the reporting of Observational Studies in Epidemiology (STROBE) Statement: guidelines for reporting observational studies. Int J Surg.

[20] Berthelsen H, Westerlund H, Bergström G, Burr H. Validation of the Copenhagen Psychosocial Questionnaire Version III and establishment of benchmarks for psychosocial risk management in Sweden. IJERPH. 2020;17(9). 10.3390/ijerph17093179 PMC7246423 32370228

[21] Burr H, Berthelsen H, Moncada S, Nübling M, Dupret E, Demiral Y (2019). The third version of the Copenhagen Psychosocial Questionnaire. Saf Health Work.

[22] O’Cathain A, Thomas KJ (2004). Any other comments?“ Open questions on questionnaires - a bane or a bonus to research?. BMC Med Res Methodol.

[23] Berthelsen H, Muhonen T, Bergström G, Westerlund H, Dollard MF (2020). Benchmarks for evidence-based risk assessment with the swedish version of the 4-Item Psychosocial Safety Climate scale. IJERPH.

[24] Hall GB, Dollard MF, Coward J (2010). Psychosocial safety climate: development of the PSC-12. Int J Stress Manag.

[25] Rosenthal LJ, Byerly A, Taylor AD, Martinovich Z (2018). Impact and prevalence of physical and verbal violence toward healthcare workers. Psychosomatics.

[26] Sauer PA, McCoy TP (2017). Nurse bullying: impact on nurses’ health. West J Nurs Res.

[27] Arnetz JE, Hamblin L, Ager J, Luborsky M, Upfal MJ, Russell J (2015). Underreporting of workplace violence: comparison of self-report and actual documentation of hospital incidents. Workplace Health Saf.

[28] Ramzi ZS, Fatah PW, Dalvandi A (2022). Prevalence of workplace violence against healthcare workers during the COVID-19 pandemic: a systematic review and meta-analysis. Front Psychol.

[29] Sahebi A, Jahangiri K, Sohrabizadeh S, Golitaleb M (2019). Prevalence of workplace violence against personnel of emergency medical services in Iran: a systematic review and meta-analysis. Iran J Psychiatry.

[30] Najafi F, Fallahi-Khoshknab M, Ahmadi F, Dalvandi A, Rahgozar M (2018). Antecedents and consequences of workplace violence against nurses: a qualitative study. J Clin Nurs.

[31] Pich J, Hazelton M, Sundin D, Kable A (2011). Patient-related violence at triage: a qualitative descriptive study. Int Emerg Nurs.

[32] Ashton RA, Morris L, Smith I (2018). A qualitative meta-synthesis of emergency department staff experiences of violence and aggression. Int Emerg Nurs.

[33] Nyberg A, Kecklund G, Hanson LM, Rajaleid K (2021). Workplace violence and health in human service industries: a systematic review of prospective and longitudinal studies. Occup Environ Med.

[34] Nayak M, Narayan K (2019). Strengths and weakness of online surveys. IOSR-JHSS.

[35] World Medical Association (2013). Declaration of Helsinki: ethical principles for medical research involving human subjects. JAMA.

